# Diagnostic accuracy of thromboelastometry and its correlation with the HPLC-MS/MS quantification test

**DOI:** 10.1590/1414-431X20198006

**Published:** 2019-04-08

**Authors:** V.F. de Aranda, P.B.M. Derogis, L.R. Sanches, C.L.P. Mangueira, M. Katz, A.C.L. Faulhaber, C.E.A. Mendes, C.E. dos Santos Ferreira, C.N. França, J.C. de Campos Guerra

**Affiliations:** 1 Hospital Israelita Albert Einstein, São Paulo, SP, Brasil; 2 Pós Graduação em Ciências da Saúde, Universidade Santo Amaro, São Paulo, SP, Brasil

**Keywords:** Anticoagulants, Blood coagulation, Plasma concentration, Rivaroxaban, Tandem mass spectrometry, Thromboelastometry

## Abstract

The aim of the study was to evaluate the diagnostic accuracy of thromboelastometry for assessing rivaroxaban concentrations. The accuracy of thromboelastometry was compared with the high-performance liquid chromatography tandem mass spectrometry (HPLC-MS/MS) method, which is the gold standard for drug plasma monitoring (the reference standard). Forty-six clinically stable patients were treated with 10, 15, or 20 mg of rivaroxaban once daily (OD group) or 15 mg twice a day (BID group) (no particular indication for treatment). Patient samples were collected 2 h after the use of the medication (peak) and 2 h before the next dose (trough). The rivaroxaban plasma concentrations were determined via HPLC-MS/MS, and thromboelastometry was performed using a ROTEM® delta analyzer. There were significant prolongations in clotting time (CT) for the 10, 15, and 20 mg of rivaroxaban treatments in the OD groups. In the 15 mg BID group, the responses at the peak and trough times were similar. At the peak times, there was a positive correlation between the plasma concentration of rivaroxaban and CT (Spearman correlation rho=0.788, P<0.001) and clot formation time (rho=0.784, P<0.001), and a negative correlation for alpha angle (rho=−0.771, P<0.001), amplitude after 5 min (rho=−0.763, P<0.001), and amplitude after 10 min (rho=−0.680, P<0.001). The CT presented higher specificity and sensitivity using the cut-off determined by the receiver characteristics curve. ROTEM has potential as screening tool to measure possible bleeding risk associated with rivaroxaban plasma levels.

## Introduction

Rivaroxaban (Xarelto®; Bayer Schering Pharma AG, Germany) is an oral anticoagulant that acts as a direct factor Xa inhibitor. This drug can be used for prevention of venous thromboembolism in hip or knee replacement surgery, in the treatment or prevention of recurrent deep vein thrombosis and pulmonary embolism, in the prevention of stroke and systemic embolism in patients with non-valvular atrial fibrillation, and in the prevention of atherothrombotic events after an acute coronary syndrome (1–3). Studies have shown a predictable and quick anticoagulant effect (within 2–4 h), with a half-life of 7–11 h and 11–13 h for young and elderly people, respectively (4 [Bibr B05]).

Rivaroxaban belongs to a new generation of oral anticoagulants (non-vitamin K antagonist oral anticoagulants) that has advantages over classic vitamin K antagonists, including predictable pharmacokinetics and pharmacodynamics, less frequent drug-drug and food-drug interactions, typically no need for laboratory monitoring, and a wide therapeutic window. The disadvantages include uncertainty regarding the assessment of drug levels, safe drug levels for major surgeries, and management of major bleeding (4–6).

In some situations, including life-threatening bleeding and urgent surgery, it is important to have assays that can correlate the plasma drug levels and hemorrhagic risk (7–8). Baglin et al. (9) presented three main methodologies for rivaroxaban monitoring: activated partial thromboplastin time (APTT), prothrombin time (PT), and determination of plasma drug levels or drug concentration. Although they are easily available and cheap, APTT and PT assays are not ideal assays for rivaroxaban measurement due to a discreet variation compared to normal values and a high dependency on the reagent used (10–13). As Thom et al. (13) reported, these tests present high specificity but lack sensitivity. In another work, our group also demonstrated this characteristic (14).

The chromogenic drug-specific anti-Xa assay and high-performance liquid chromatography tandem mass spectrometry (HPLC-MS/MS) can be used for plasma quantitation. Bardy et al. (15) cited the main advantages for the use of chromogenic methods: accessibility, good reproducibility, and repeatability; ability to be carried out in less than 5 min; and linearity over a wide concentration range. Nevertheless, these methods carry some disadvantages. The most cited are the lower limit of quantification (20 ng/mL) and the sample opacity, e.g., icteric, lipemic, and/or hemolyzed samples can interfere with these methods (16). These limitations support the increasing interest in the development of HPLC-MS/MS methods. In a previous work, we showed the ability of HPLC-MS/MS to quantify plasma rivaroxaban levels (17).

Several HPLC-MS/MS methods for rivaroxaban plasma level determination show that this technique combines high accuracy, low matrix effect, and high sensitivity for drug monitoring (18–20). Nevertheless, the increased turnaround time can compromise the assay’s utility in an emergency setting, where a point-of-care (POC) method may provide a faster result. Additionally, according to Grebe and Singh (21), LC-MS/MS methods have limitations: highly manual workflows, complex operation and maintenance, sample throughput limits, insufficient detection sensitivity for some analytes, and problems with detection specificity.

The main advantage of the POC technique is the ability to move the testing closer to the patient. ROTEM® (rotational thromboelastometry) is a POC viscoelastic coagulation test that allows rapid detection of coagulation abnormalities (22,23). This POC test is currently used for patient hemostatic monitoring during and after cardiac surgery, peripartum hemorrhage (24), and liver transplantation (25,26), and has been previously evaluated for anticoagulant therapy monitoring (7,27,28).

The present study aimed to evaluate the effect of variable rivaroxaban dosing on thromboelastometry and its correlation with plasma concentrations as determined by HPLC-MS/MS.

## Material and Methods

### Population

Forty-six patients were admitted to a Hospital of High Complexity and were treated with 10, 15, or 20 mg of rivaroxaban daily or 15 mg twice a day. The inclusion criteria were as follows: age of 18 years or older, no oncologic disease and normal renal function, currently on rivaroxaban treatment (no specific indication for treatment), and no use of drugs that alter platelet function or other anticoagulant drugs. The exclusion criteria were pregnancy and age under 18 years.

Signed informed consent was required from each patient in accordance with the Declaration of Helsinki and ethical protocol regulations. The study was approved by the local Ethical Research Committee under CAAE number: 43080215.9.0000.0071 (Albert Einstein Israeli Hospital Ethical Committee).

### Samples

Blood was collected into citrated plastic tubes (Sarstedt, Germany) 2 h before (trough) and 2 h after drug intake (peak). Whole blood was subjected to thromboelastometry. Plasma was separated after centrifugation (2250 *g* for 15 min at 10°C) and stored at –80°C until analysis.

Sample processing was performed by protein precipitation with methanol, as described previously (17). Briefly, methanol (400 μL) containing deuterated internal standard (IS) (rivaroxaban-d4, 500 ng/mL) was added to 200 μL of deproteinated samples that were centrifuged at 1800 *g* for 10 min at 4°C. The supernatants were filtered through a 0.22-μm PVDF filter (Millex®-GV, Merck Millipore, Ireland) and transferred to an amber clean autosampler vial with insert for analysis; 2 μL was injected into the HPLC-MS/MS system to determine the rivaroxaban levels.

### Thromboelastometry

Whole blood samples were analyzed by rotational thromboelastometry using a ROTEM delta (TEM Innovations GmbH; Germany) analyzer within 2 h of acquisition. Non-activated thromboelastometry (NATEM) tests were carried out in accordance with the manufacturer's recommendations using disposable cups and pins (Cup and Pin Pro, TEM Innovations GmbH, Germany). The automated pipetting system was used to recalcify and activate the 300 μL blood with 20 μL STARTEM reagent (0.2 mol/L CaCl_2_ in HEPES buffer pH 7.4 and 0.1% sodium azide - TEM Innovations GmbH, REF: 503-10). CT (s), CFT (s), alpha angle, and maximum clot firmness (MCF, mm) amplitudes after 5 and 10 min (mm) were analyzed (1,29).

### HPLC-MS/MS method

Chromatography was performed on an Agilent 1260 LC system (Agilent Technologies, Canada) (17). The compounds were eluted from a Kinetex C18 HPLC column (100×3 mm, 2.6-μm particle size; Phenomenex, USA) in an isocratic gradient. The flow rate and column temperature were set at 0.5 mL/min and 40°C, respectively. The LC system was coupled to an SCIEX QTRAP 5500 tandem mass spectrometer (SCIEX, Canada) fitted with an electrospray ionization (ESI) source.

### Statistical analysis

Comparisons between groups were done using the Mann-Whitney test. Correlations between thromboelastometric parameters and rivaroxaban plasma concentrations measured by HPLC-MS/MS were evaluated by the Spearman rank correlation coefficient (rho).

The diagnostic test was evaluated using the free online MEDCALC easy-to-use statistical software and SPSS Statistics 21.0 (IBM, USA). Sensitivity was defined as the percentage of samples with plasma concentrations >30 ng/mL that were correctly identified as samples with hemorrhagic risk. Correspondingly, specificity was defined as the percentage of samples with plasma concentrations <30 ng/mL that were correctly identified without the risk. Sensitivity/specificity >95% was defined as sufficient for clinical application. Confidence interval for sensitivity and specificity were “exact” Clopper-Pearson CI; for the predictive values, the standard logit CI given by Mercaldo et al. (30) was used. A plasma concentration of 30 ng/mL was selected as cut-off defined as safe for invasive procedures (31). Receiver operating characteristic (ROC) curves were used to assess the ability of ROTEM® parameters to identify patients with hemorrhagic risk associated with plasma concentration. The area under the ROC curve (AUC) was provided as an overall measure of test performance. An AUC <0.6 was considered a failure to predict hemorrhagic risk associated with plasma concentration. Optimal cut-off values were identified with a Youden index and were presented along with their respective sensitivities and specificities [95% confidence interval (CI)].

Statistical analysis was performed using GraphPad Prism 5 (GraphPad Software, USA), and significance was set at P<0.05.

## Results

### Patient descriptions

This study evaluated the concentration of rivaroxaban in plasma samples by HPLC-MS/MS and coagulation parameters by thromboelastometry in a group of 46 patients. These patients were between 25 and 95 years old, and 48% were female (Table 1). All received oral rivaroxaban anticoagulant doses between 10 and 20 mg. Thirteen patients received 10 mg of rivaroxaban OD, 16 patients were treated with 15 mg of rivaroxaban OD, 8 patients were treated with 15 mg of rivaroxaban BID, and 9 patients were treated with 20 mg of rivaroxaban OD.

**Table 1:** Demographic data.

Gender, n (%)	
Female	22 (48)
Male	24 (52)
Rivaroxaban dosage, n (%)	
10 mg, OD	12 (28)
15 mg, OD	16 (35)
15 mg, BID	8 (17)
20 mg, OD	9 (20)
Age (years)	
Average (standard deviation)	69 (21)
Minimum and maximum age	25-95

OD: once daily; BID; twice daily.

### Plasma concentration by HPLC-MS/MS

All 92 plasma samples were submitted to plasma concentration determination using the HPLC-MS/MS method. As expected, there were significant differences between the trough and peak levels for OD therapy (Supplementary Table S1).

### Thromboelastometric parameters

**Figure 1:**
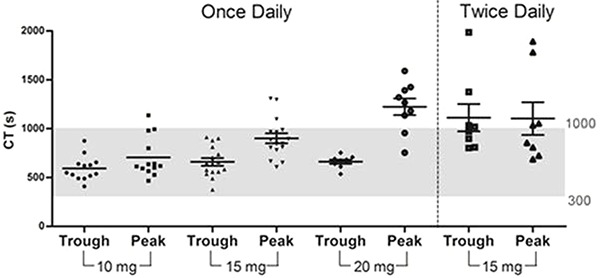
Clotting times (CT) in the groups evaluated.

A summary of the thromboelastometric results is presented in Supplementary Table S1. There were significant differences between trough and peak responses of CT, CFT, alpha angle, and A5 results for 15 and 20 mg of rivaroxaban OD. For 10 mg of rivaroxaban OD, it was possible to differentiate trough from peak using the CT parameter. For A10, it was possible to differentiate trough and peak only in the group receiving 20 mg of rivaroxaban OD. Furthermore, as shown in Supplementary Table S1 and Figure 1, the CT parameter presented an increase in mean response in peak levels in a dose-dependent manner; for 20 mg, the mean value to peak level was out of normal range (Supplementary Table S2). For the group receiving BID therapy, it was clear that there were no differences between the trough and peak times.

### Correlation between HPLC-MS/MS and thromboelastometry

**Figure 2:**
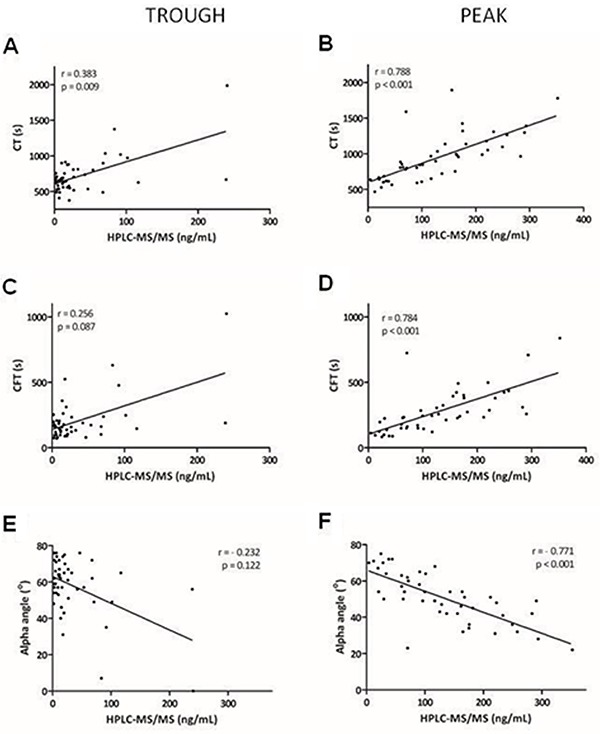
Correlation between high-performance liquid chromatography tandem mass spectrometry (HPLC-MS/MS) and thromboelastometry differentiating trough and peak for clotting time (CT) (**A** and **B**), clot formation time (CFT) (**C** and **D**), and alpha angle (**E** and **F**) (n=92).

**Figure 3:**
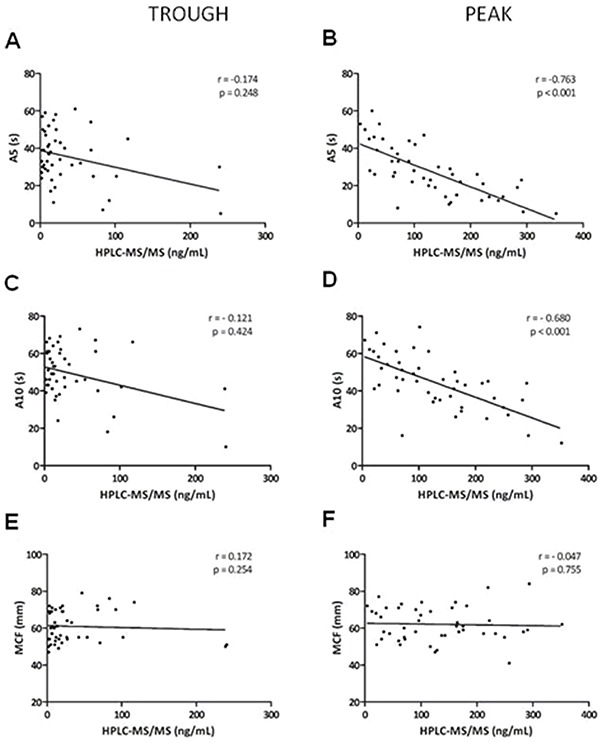
Correlation between high-performance liquid chromatography tandem mass spectrometry (HPLC-MS/MS) and thromboelastometry differentiating trough and peak for A5 (**A** and **B**), A10 (**C** and **D**), and maximum clot firmness (MCF) (**E** and **F**) (n=92).

We compared the plasma concentration of rivaroxaban by HPLC-MS/MS and thromboelastometry at trough and peak times. At peak time, there was a significantly positive correlation between the plasma concentration of rivaroxaban and both the clotting time (Spearman correlation rho=0.788, P<0.001, Figure 2B) and the clot formation time (rho=0.784, P<0.001, Figure 2D), and a negative correlation with the alpha angle (rho=−0.771, P<0.001, Figure 2F), A5 (rho=−0.763, P<0.001, Figure 3B), and A10 (rho=−0.680, P<0.001, Figure 3D). No correlation was observed between the maximum firmness of the clot and the plasma concentration of the drug at the trough and peak times (Figures 3E and F).

### ROTEM diagnostic accuracy

**Figure 4:**
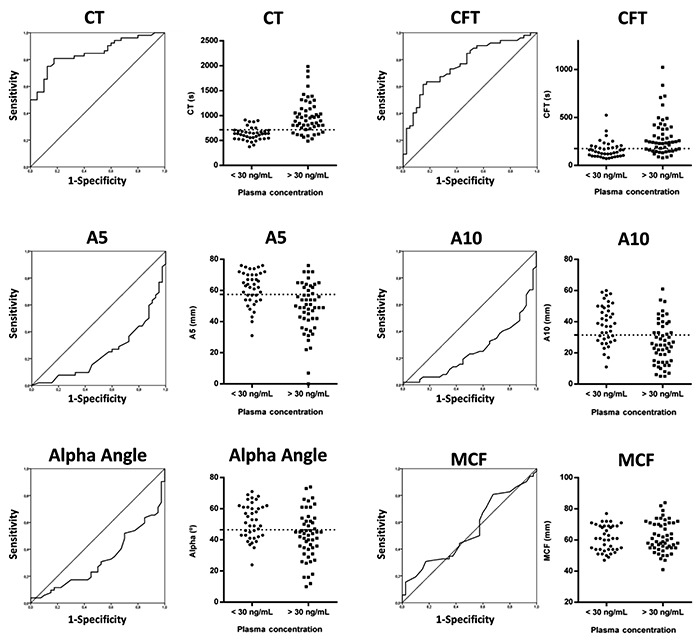
ROC curves of ROTEM parameters predicting rivaroxaban plasma concentration associated to a hemorrhagic risk in emergency cases. CT: clotting time CFT: clot formation time; MCF: maximum clot firmness.

Rivaroxaban concentration was <30 ng/mL in 38/92 samples. Based on ROC curves (Figure 4 and Table 2), only CT and CFT were good predictors of rivaroxaban plasma concentration >30 ng/mL, presenting an AUC of 0.85 (0.77−0.93) and 0.77 (0.67−0.86), respectively.

**Table 2:** Diagnostic accuracy for ROTEM parameters using NATEM reagent.

Parameter	AUC (95%CI)	Optimal cut-off	Sensitivity %, (95%CI)	Specificity %, (95%CI)	Positive predictive value %, (95%CI)	Negative predictive value %, (95%CI)
CT	0.85 (0.77−0.93)	715	80.8 (67.5−90.4)	82.5 (67.2−92.7)	85.7 (75.1−92.3)	76.7 (65.0−85.4)
CFT	0.77 (0.67−0,86)	176	69.2 (54.9−81.3)	65.0 (48.3−79.4)	72.0 (61.9−80.3)	61.9 (50.5−72.2)
A5	0.25 (0.15−0.35)	57.5	71.2 (56.9−82.9)	65.0 (48.3−79.4)	72.6 (62.6−80.7)	63.4 (51.7−73.8)
A10	0.26 (0.16−0.35)	31.5	67.3 (52.9−79.7)	65.0 (48.3−79.4)	71.4 (61.2−79.9)	60.5 (49.4−70.6)
Alpha angle	0.32 (0.21−0.43)	46.5	65.4 (50.9−78.0)	62.5 (45.8−77.3)	69.4 (59.2−78.0)	58.1 (47.1−68.4)
MCF	0.54 (0.42−0.66)					

AUC: area under the ROC curve; CT: clotting time CFT: clot formation time; MCF: maximum clot firmness.

The calculated cut-off value of CT was 715 s (sensitivity: 80.8%, specificity: 82.5%) and CFT was 176 s (sensitivity: 69.2%, specificity: 65.0%). CT presented the best AUC (0.85 [0.77−0.93]) followed by CFT (0.77 [0.67−0.86]) (Table 2).

Furthermore, the MCF parameter was insensitive to rivaroxaban plasma level. A5, A10, and alpha angle were not able to predict the rivaroxaban plasma concentration >30 ng/mL, the AUCs were below 0.5.

## Discussion

This was a validation of a laboratory method that may be useful in assessing the risk of bleeding in patients taking rivaroxaban. There is currently no specific test available 24 h for this purpose. In our service, thromboelastometry is routinely used to evaluate patients with severe hemorrhagic disease and highly complex surgeries.

Recently, rivaroxaban has expanded its clinical indications. Currently, various doses, administration frequencies, and treatment durations are defined for each therapeutic indication (2). Because of its predictable pharmacology, rivaroxaban does not require therapeutic monitoring, except in specific cases including emergency, urgent surgery, and bleeding. Although the HPLC-MS/MS method allows accurate measurement in a wide range of plasma concentrations, this test is time-consuming and can be difficult to use in emergency situations, although it can be applicable in diverse types of matrices (17). Recently, POC methodologies such as ROTEM tests are gaining interest for anticoagulant monitoring (32), because of the rapid detection of coagulation abnormalities at POC (7).

In this context, this study compared the plasma concentration of rivaroxaban, a direct inhibitor of factor Xa, in individuals taking doses ranging from 10 to 20 mg (OD or BID), as determined by HPLC-MS/MS and the parameters of thromboelastometry determined by the ROTEM test, showing rapid changes in the coagulation processes. Despite the high interindividual variation in the plasma concentrations *vs* the rivaroxaban therapeutic regimen (33), it was possible to differentiate peak and trough by HPLC-MS/MS and by thromboelastometric parameters CT, CFT, alpha angle, and A5 for 10, 15, and 20 mg of rivaroxaban OD regimens.

Our results for rivaroxaban plasma concentrations concerning the mean peak and trough levels were similar to those described by Mueck et al. (34). Peak levels are all associated with hemorrhagic risk, since the normal range values described are higher than the cut-off of 30 ng/mL. Trough reference values vary from a safe region (plasma concentration <30 ng/mL) to values associated with hemorrhagic risk (concentration >30 ng/mL), and for this reason, measuring the rivaroxaban plasma concentrations is relevant.

This study was conducted using patient plasma samples. Lim et al. (35) conducted a review to demonstrate the differences between reagent sensitivity depending on whether spiked or patient samples are used. As example, they cited some studies that showed that the use of lyophilized plasma may prolong values of the traditional coagulation tests PT and APTT beyond those of fresh plasma.

Our findings were compatible with those of Casutt et al. (1), and the observed differences could be due to the sensitivity to the reagents (Intem and Extem *vs* Natem). The authors showed significantly prolonged CT (using Extem and Intem), CFT (Extem), and alpha angle (Extem) values. These differences between reagents were also found by Chojnowski et al. (7) and Schenk et al. (3). Extem is a specific reagent for assessing the extrinsic coagulation pathway. The conventional coagulation parameter (PT) is also specific for the extrinsic coagulation pathway and is a standard assay for monitoring rivaroxaban (9). Natem is sensitive for assessing the equilibrium of coagulation activation or inhibition. Its specificity is lower, possibly the main reason for the differences found.

According to Schenk et al. (3), in a group taking 15 or 20 mg of rivaroxaban OD, rivaroxaban-dependent prolongation increases in CT were observed in patients using Extem, Intem, and Fibtem. In another study, Tsantes et al. (36) used the Natem® reagent and, at the peak time (3 h after taking 20 mg of rivaroxaban), showed prolongation of the CT and CFT parameters for 20 patients with non-valvular atrial fibrillation beyond those of a control group. The MCF parameter was not influenced by the presence of the drug. Our findings at the peak time are consistent with their results. The prolongation of CT in response to rivaroxaban was also found *in vitro* by Korber et al. (37). The MCF parameter is dependent on platelets, fibrinogen, and coagulation factors. In clinical events related to hemostatic defects, such as cirrhosis, the MCF may be used to measure the bleeding risk (38).

The CFT results obtained in our study are in accordance with those obtained by Tsantes et al. (36), although they seem contradictory to those presented by Chojnowski et al. (7), who evaluated the effects of rivaroxaban therapy on ROTEM coagulation parameters in patients with venous thromboembolism in a group of 30 patients taking 20 mg/day of rivaroxaban using four different reagents: Extem, Intem, Fibtem, and Aptem. They compared the peak and trough levels to the results from a control group and observed no significant difference between patients before rivaroxaban dosing and controls. They also identified a better differentiation between peak and trough times using CT. In addition, they determined that the ROTEM tests CFT and MCF were insensitive to rivaroxaban. These discrepancies probably result from the sensitivity of reagents used. They used specific reagents for extrinsic and intrinsic coagulation pathways, ETEM and INTEM and we used NATEM. On the other hand, the same authors found a behavior for the CT parameter similar to that of our findings, using two different types of reagents (Extem and Intem) versus plasma concentration, as determined by the anti-Xa assay. Evidence of a significant correlation between drug concentration and thromboelastometric parameters (CT, CFT, alpha angle, A5, and A10) was observed only after rivaroxaban administration (peak).

During the trough time, it is possible that the ROTEM parameters may tend to normalize to baseline, as showed by Bowry et al. (39), compared to the thromboelastographic (TEG) parameters that may be responsible for the lower correlation observed. The TEG® and ROTEM® are similar technologies, as described by Sankarankutty et al. (29) and Dias et al. (40).

Considering a potential use in emergency situations, the diagnostic accuracy for ROTEM parameters using the cut-off of 30 ng/mL was also studied. Pernod et al. (31) and Ebner et al. (32) described these limits as safe for invasive procedures. Our results suggest that the main ROTEM parameters may be used as predicting tools for rivaroxaban plasma concentration. The diagnostic accuracy is totally dependent on cut-off parameters. Using the clinical normal range of the CT parameter (300–1000 s), the positive predictive value rose to 100% accompanied by a significant loss of specificity (data not shown).

The main limitations of our study include: sample size, the dosing regimens, the use of only one reagent in thromboelastometry, and the selection of stable patients. A small number of patients for each therapeutic regimen was analyzed. It was not possible to study patients taking 2.5 mg of rivaroxaban OD. The conclusions generated by our results should be further investigated in a larger group of patients and with various types of thromboelasmetric reagents.

In conclusion, the oral intake of rivaroxaban produced a statistically significant impairment of most of the ROTEM parameters measured after 2 h compared to the trough time, in a dose-dependent manner. Despite the limitations of ROTEM in measuring the response to rivaroxaban at the trough time, it does present some advantages that make it possible to obtain quick results. The analysis using ROTEM has a series of parameters that together describe the dynamic formation, stabilization, and dissolution of the clot. These parameters provide different information of the same plasma concentration that may be complementary. The CT parameter is a sensitive and specific tool for predicting the concentration associated with hemorrhage risk and the A5, for example, to predict the opposite response. All these observations can be obtained in a single analysis, in a short time, corroborating the clinical potential of Rotem.

## Supplementary material

Click here to view [pdf].
